# Associations of Serum 25(OH)D Concentrations with Lung Function, Airway Inflammation and Common Cold in the General Population

**DOI:** 10.3390/nu10010035

**Published:** 2018-01-03

**Authors:** Rachida Rafiq, Willemien Thijs, Robert Prein, Renate T. de Jongh, Christian Taube, Pieter S. Hiemstra, Renée de Mutsert, Martin den Heijer

**Affiliations:** 1 Department of Internal Medicine and Endocrinology, VU University Medical Center, Amsterdam Movement Sciences, 1081 HV Amsterdam, The Netherlands; rt.dejongh@vumc.nl (R.T.d.J.); m.denheijer@vumc.nl (M.d.H.); 2 Department of Pulmonology, Leiden University Medical Center, 2333 ZC Leiden, The Netherlands; willemienthijs@hotmail.com (W.T.); raprein@gmail.com (R.P.); christian.taube@rlk.uk-essen.de (C.T.); p.s.hiemstra@lumc.nl (P.S.H.); 3 Department of Pulmonology, Haaglanden Medisch Centrum, 2501 CK Den Haag, The Netherlands; 4 Department of Pulmonary Medicine, Ruhrlandklinik, West German Lung Center, University Hospital Essen, 457147 Essen, Germany; 5 Department of Clinical Epidemiology, Leiden University Medical Center, 2333 ZC Leiden, The Netherlands; r.de_mutsert@lumc.nl

**Keywords:** vitamin D, lung function, pulmonary function, airway inflammation, airway infection, cold, obesity

## Abstract

Vitamin D is hypothesized to have a beneficial effect on lung function and respiratory infections. The aim of this study was to assess the relationship of serum 25-hydroxyvitamin D (25(OH)D) concentrations with lung function, airway inflammation and common colds. We performed a cross-sectional analysis in the Netherlands Epidemiology of Obesity (NEO) study, a population-based cohort study. We included participants with measurements of serum 25(OH)D, Forced Expiratory Volume in 1 s (FEV_1_), Forced Vital Capacity (FVC), Fractional Exhaled Nitric Oxide (Fe_NO_), and data on self-reported common colds (*n* = 6138). In crude associations, serum 25(OH)D was positively associated with FEV_1_ and FVC, and negatively with Fe_NO_ and the occurrence of a common cold. After adjustment for confounders, however, these associations disappeared. Stratified analyses showed that Body Mass Index (BMI) was an effect modifier in the relationship between serum 25(OH)D and FEV_1_, FVC and Fe_NO_. In obese participants (BMI ≥ 30 kg/m^2^), 10 nmol/L higher 25(OH)D was associated with 0.46% predicted higher FEV_1_ (95% Confidence Interval: 0.17 to 0.75), 0.46% predicted higher FVC (0.18 to 0.74), and 0.24 ppb lower Fe_NO_ (−0.43 to −0.04). Thus, in the total study population, 25(OH)D concentrations were not associated with lung function, airway inflammation and common colds. In obese participants, however, higher 25(OH)D concentrations were associated with a better lung function and lower airway inflammation.

## 1. Introduction

The role of vitamin D in bone mineralization and calcium homeostasis is well established [1]. In addition, there is a large amount of evidence supporting the influence of vitamin D on immune function and inflammatory disease [2]. In particular, there has been interest in the role of vitamin D in respiratory outcomes such as lung function and respiratory infections [3,4,5,6,7,8,9,10].

In the past decade, several observational studies examined the relationship of vitamin D status with lung function in the general population, leading to inconsistent results, with some studies finding a relationship [4,5], and others not [6,7]. Vitamin D status has also been studied in patients with Chronic Obstructive Pulmonary Disease (COPD) and asthma specifically, where it has been associated with disease severity [11,12]. The exact mechanisms by which vitamin D affects lung function are unknown, but it has been hypothesized that effects of vitamin D on tissue remodeling, muscle function and/or airway inflammation may play a role [13,14].

The role of vitamin D in the immune system has been extensively studied in vitro and vitamin D has been hypothesized to have a dual effect. First, vitamin D decreases inflammatory reactions through the inhibition of NF-κB-pathways [15]. Second, vitamin D improves antimicrobial defense by inducing the production of antimicrobial peptides, and increasing antibacterial and antiviral defenses [16,17,18]. In observational studies, it has been shown that low vitamin D status is inversely associated with number of respiratory tract infections [5,8,9,10]. In particular in asthma and COPD patients, respiratory tract infections play an important role as they are associated with exacerbations [19,20], which are the main cause of disease progression, morbidity and mortality in these patients [21,22]. In addition, vitamin D status has been inversely related to measures of airway inflammation in children with asthma [23]. This may suggest that vitamin D deficiency is a risk factor for respiratory infections and inflammation. In two intervention trials in patients with COPD, vitamin D supplementation was shown to decrease exacerbation rate, but only in participants with vitamin D deficiency at baseline [24,25].

Previous studies have shown that the relationship of vitamin D status with lung function and airway inflammation might be affected by sex and adiposity. In an ageing cohort, vitamin D status was only associated with peak expiratory flow (PEF) in men, but not in women [26]. In another study, associations were stronger in subgroups of participants with a Body Mass Index (BMI) of ≥25 kg/m^2^ compared to subgroups with a BMI < 25 kg/m^2^ [27]. These differences might explain the inconsistent results of previous studies. Our aim was to study the associations of serum 25-hydroxyvitamin D (25(OH)D) concentrations with lung function, airway inflammation and the occurrence of a common cold, and whether associations differed between men and women, or different BMI groups. We hypothesized that low serum 25(OH)D concentrations are associated with an impaired lung function and increased airway inflammation. Furthermore, we hypothesized that low serum 25(OH)D concentrations are associated with a higher number of recent common colds.

## 2. Materials and Methods 

### 2.1. Study Design and Study Population 

The Netherlands Epidemiology of Obesity (NEO) study is a population-based prospective cohort study in 6671 men and women aged between 45 and 65 years included between September 2008 and September 2012. The present study is a cross-sectional analysis of the baseline measurements.

Design and data collection of the study has been described in detail previously [28]. Men and women with self-reported BMI ≥ 27 kg/m^2^ living in the greater area of Leiden (in the west of the Netherlands) were eligible to participate in the NEO study. In addition, in one municipality (Leiderdorp), all inhabitants aged 45 to 65 years were invited, irrespective of their BMI, allowing for a reference distribution of BMI. Participants were invited for a baseline visit at the NEO study center of the Leiden University Medical Center (LUMC) after an overnight fast. Prior to this study visit, participants completed a general questionnaire at home to report demographic, lifestyle and clinical information. All participants underwent an extensive physical examination, including blood sampling and spirometry. In the present analysis, we excluded subjects with missing data. The study was approved by the medical ethics committee of the Leiden University Medical Center (LUMC) and all participants gave written informed consent.

### 2.2. Data Collection

On the questionnaires, participants reported their medical history, including asthma and COPD, and use of medication, including pulmonary and anti-inflammatory medicine. Ethnicity was assessed by self-identification in eight categories, which were grouped into ‘white’ and ‘other’. Self-reported education was grouped as low versus high education. Tobacco smoking was reported in three categories: ‘current smoker’, ‘former smoker’ and ‘never smoker’. In addition, the number of pack-years was calculated. Participants also reported when they had had a common cold for the last time, to which they could answer with: ‘within a week’, ‘between 1 week and 1 month ago’, ‘between 1 and 6 months ago’, ‘between 6 months and a year ago’ or ‘more than 1 year ago’. For the analyses, this was dichotomized into ‘within a month’ and ‘more than a month ago’. Participants reported the frequency and duration of their physical activity during leisure time using the Short Questionnaire to assess health-enhancing physical activity. This was expressed in Metabolic Equivalent of Task-hours (MET-hours) per week [29].

### 2.3. 25(OH)D Measurements

At the baseline study visit, a fasting blood sample of venous blood was collected by venipuncture, and immediately sent to the central clinical laboratory of the LUMC for the assessment of serum 25-hydroxyvitamin D (25(OH)D) concentrations. During the inclusion period of the NEO study, quantification of the 25(OH)D concentration in the serum was done by three sequential methods. From 1 September 2008 to 4 October 2010, the radioimmunoassay (RIA) method was used (DiaSorin, Saluggia, Italy). From 5 October 2010 to 29 September 2011, the Chemoluminescent Immunoassay was used (iSYS analyzer, ImmunoDiagnostics Inc., Boldon, UK). Finally, from 30 September 2011 until the end of the study, the 2nd generation Electrochemoluminescence Immunoassay (ECLIA) (Modular Analytics E170 analyzers, Roche Diagnostics, Mannheim, Germany) was used. Two-level commercial Internal Quality Control (IQC) samples were used in all three methods to monitor performance. Maximum overall CVa was <12%. All methods have stated specificity for both 25-hydroxyvitamin D_2_ and D_3_ (25(OH)D_2_ and 25(OH)D_3_).

Because three different immunoassays were used during the study period, serum 25(OH)D was calibrated towards the “golden standard” liquid chromatography/tandem mass spectrometry (LC-MS/MS) method (isotope dilution/online solid-phase extraction liquid chromatography/tandem mass spectrometry (ID-XLC-MS/MS)) to minimize possible variations. These LC-MS/MS measurements were performed at the Endocrine Laboratory of the VU University Medical Center (Amsterdam, The Netherlands) as described before [30]. The limit of quantitation (LOQ) was 4.0 nmol/L; intra-assay CV was <6%, and inter-assay CV was <8% for concentrations between 25 and 180 nmol/L. 25(OH)D_2_ and 25(OH)D_3_ were measured separately. From measurements of each of the three different 25(OH)D assays used, 50 samples were selected to determine serum 25(OH)D with LC/MS-MS. Previous studies have shown that 50 samples suffice to fit an equation for comparison between the different assays [31]. Samples were selected according to tentiles of serum 25(OH)D within each of the methods used. Within each tentile, 5 samples were selected at intervals during the period in which the method was used. This time-dependent sampling was added to minimize the contribution of inter-assay variation to variability between the different assays. Calibrated serum 25(OH)D concentrations were then calculated using linear regression formulas.

### 2.4. Lung Function Assessments

All participants of the NEO study underwent spirometry at the Pulmonology department of the LUMC. The Forced Expiratory Volume in 1 s (FEV_1_) and Forced Vital Capacity (FVC) were determined. Participants were required to perform at least three reproducible forced expiratory maneuvers, with a maximum difference of 5 percent or 150 mL between the highest and lowest measurement. Of these three maneuvers, the one with the highest value of FEV_1_ and FVC together was used in the analyses [32].

### 2.5. Fractional Exhaled Nitric Oxide

Fractional Exhaled Nitric Oxide (Fe_NO_) was measured using a portable analyzer, the NIOX MINO (Aerocrine AB, Solna, Sweden). Participants performed a 10-s slow steady exhalation. Our previously published results demonstrate that, in large cohorts of overweight and obese adults, a single exhaled NO measurement suffices, and therefore one recording expressed as parts per billion (ppb) was made [33].

### 2.6. Body Composition Measures

At the study site, height and weight were measured with precision of 0.1 cm/kg. BMI was calculated by dividing the weight in kilograms by the height in squared meters. For stratification, BMI was categorized into three categories: <25, 25–30 and ≥30 kg/m^2^ according to WHO criteria [34]. Waist circumference was measured between the border of the lower costal margin and the iliac crest with the precision of 0.1 cm. Total body fat (TBF) was estimated by bio-electrical impedance analysis (BIA) using the Tanita foot-to-foot BIA system TBF-300A Body Composition Analyzer (Tanita Corporation of America, Inc., Arlington Heights, IL, USA) [35].

### 2.7. Statistical Analyses

Data were analyzed using STATA version 13.1 (StataCorp LP, College Station, TX, USA). In the NEO study, there is an oversampling of persons with BMI ≥ 27 kg/m^2^. To correctly represent associations in the general population, adjustments were made for the oversampling of individuals with a BMI ≥ 27 kg/m^2^ [36]. This was done by weighting individuals towards the BMI distribution of participants from the Leiderdorp municipality whose BMI distribution was similar to the BMI distribution in the general Dutch population and could serve as a reference population [37]. Using the BMI distribution of this reference population, we calculated weight factors for the NEO population, resulting in a higher weight factor for participants with a lower BMI. Using these weight factors, we weighted all our analyses towards the BMI distribution of the general population [38]. Consequently, results apply to a population-based study without oversampling of persons with a BMI ≥ 27 kg/m^2^.

We summarized the baseline characteristics as mean (standard deviation, SD) for normally distributed continuous variables, median (interquartile range) for skewed continuous variables and percentages for categorical variables, stratified by categories of serum 25(OH)D concentrations (serum 25(OH)D <50 nmol/L, 50–75 nmol/L and ≥75 nmol/L [2]). Linear regression analyses were used to examine the associations between serum 25(OH)D and FEV_1_, FVC and Fe_NO_. Logistic regression analyses were used to calculate the odds ratio (OR) of the occurrence of a common cold in the preceding month per 10 nmol/L serum 25(OH)D.

The crude regression models were adjusted for age, sex, ethnicity, packyears of smoking, self-reported obstructive pulmonary disease, use of pulmonary and anti-inflammatory medication, educational level, season, physical activity, BMI, waist circumference and total body fat. Because serum 25(OH)D concentrations follow a sinusoidal pattern throughout the year, adjustment for season was performed using a cosinor model [39]. Potential effect modification by sex, age and BMI was examined by performing regression analyses stratified for sex, age and BMI categories. Finally, a sensitivity analysis was performed in participants with 25(OH)D concentrations <50 nmol/L.

## 3. Results

After exclusion of participants with missing data, we included 6138 participants in our analyses. The characteristics of the study population stratified by serum 25(OH)D categories are shown in Table 1. Of the total study population, 20% had serum 25(OH)D concentrations lower than 50 nmol/L. Participants with lower serum 25(OH)D concentrations (<50 nmol/L) more often had asthma and COPD, a higher BMI, total body fat and waist circumference, compared to participants with higher serum 25(OH)D concentrations. In addition, they had higher FEV_1_ and FVC, and more often reported a common cold in the preceding month.

**Figure 1:**
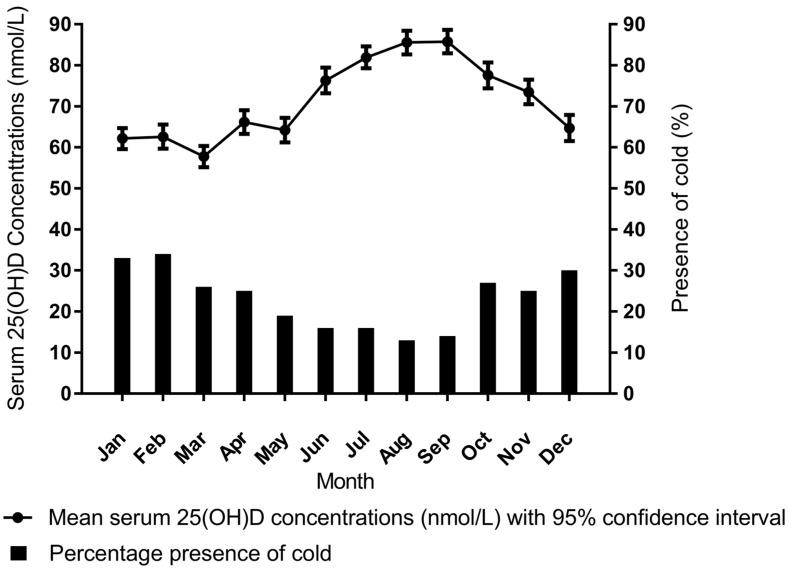
Mean serum 25(OH)D concentrations and percentage of participants that reported a recent common cold per month, in men and women participating in the Netherlands Epidemiology of Obesity study, aged between 45 and 65 years. Data are presented as mean (95% confidence interval) and percentage. Results are based on analyses weighted towards the BMI distribution of the general population (*n* = 6138). Results are shown per month, combined over different years.

In Figure 1, we have plotted the mean serum 25(OH)D concentrations and percentage of participants that reported a recent common cold, per month. Both serum 25(OH)D concentrations and the occurrence of a common cold showed a sinusoidal pattern, with an inverse relationship between serum 25 (OH) D concentrations and the occurrence of a common cold.

The results of the regression analyses are shown in Table 2. In the crude associations, serum 25(OH)D was positively associated with FEV_1_ and FVC, and negatively associated with Fe_NO_ and the occurrence of a common cold in the preceding month. In addition, 10 nmol/L higher serum 25(OH)D was associated with 0.48% predicted higher FEV_1_ (95% CI: 0.23 to 0.73), 0.83% predicted higher FVC (0.58 to 1.07) and 0.18 ppb lower Fe_NO_ (−0.39 to 0.03). The OR of the occurrence of a common cold in the preceding month was 0.94 (0.90 to 0.98) per 10 nmol/L serum 25(OH)D. After adjustment for all confounding factors, however, these associations largely disappeared. Adjustment for season attenuated the regression coefficients of FEV_1_ with 40%, of FVC with 27%, and of Fe_NO_ with 43%. Relationship with common colds was attenuated with 3%. A sensitivity analysis in participants with 25(OH)D concentrations <50 nmol/L did not show an association (Supplementary Table S1). An analysis in participants using vitamin D and multivitamin supplements only, also did not show different results (Supplementary Table S2).

**Figure 2:**
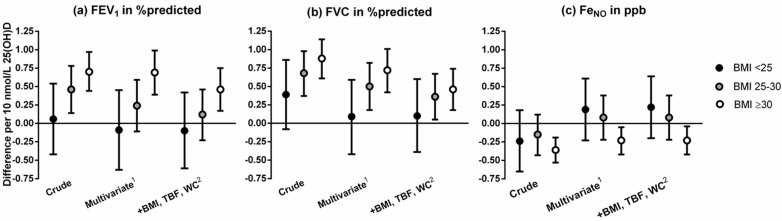
Associations of serum 25(OH)D (per 10 nmol/L) with (**a**) FEV_1_, (**b**) FVC and (**c**) Fe_NO_ stratified by Body Mass Index (BMI) category, in men and women participating in the Netherlands Epidemiology of Obesity study, aged between 45 and 65 years. Results were based on analyses (*n* = 6138) weighted towards the BMI distribution of the general population, and were derived from regression coefficients with 95% confidence intervals from linear regression analyses and expressed as difference in outcome measure per 10 nmol/L 25(OH)D stratified by BMI category (BMI < 25: 43%, BMI 25–30: 41% and BMI ≥ 30: 16%). ^1^ Multivariate: Adjusted for age, sex, ethnicity, number of packyears, self-reported obstructive pulmonary disease, use of pulmonary and anti-inflammatory medication, educational level, season and physical activity. ^2^ Multivariate plus adjustments for BMI, total body fat and waist circumference. FEV_1_: Forced Expiratory Volume in 1 s; FVC: Forced Vital Capacity; Fe_NO_: fractional exhaled nitric oxide; ppb: parts per billion; BMI: Body Mass Index; TBF: total body fat; WC: waist circumference.

After stratification for BMI categories, the associations of serum 25(OH)D with FEV_1_, FVC and Fe_NO_ differed per BMI category (Figure 2, Supplementary Table S3). Because BMI was an effect modifier in these relationships, we subsequently stratified all models by BMI (BMI < 25: 43%, BMI 25–30: 41% and BMI ≥ 30: 16% of study population). In participants with obesity (BMI ≥ 30), serum 25(OH)D was positively associated with FEV_1_ and FVC, and negatively associated with Fe_NO_. In participants with a BMI < 25, serum 25(OH)D was not associated with these outcomes after adjustment for confounding factors. Nevertheless, the stratified results showed a dose–response trend with stronger associations in higher BMI categories. No differences were found between the sexes and different age categories (Supplementary Tables S4 and S5).

## 4. Discussion

In this study, we assessed the relationship between serum 25(OH)D concentrations and lung function, fractional exhaled nitric oxide and common colds in a population-based cohort study. Whereas there were no associations in the total population, we observed that higher serum 25(OH)D concentrations were associated with a better lung function and lower airway inflammation in participants with a BMI ≥ 30, but not in participants with a BMI < 30. Serum 25(OH)D concentrations were not associated with the occurrence of common colds in the last month.

Several previous observational studies have assessed the relationship between vitamin D and lung function. Results of these studies have been inconsistent. In some studies, a positive association between vitamin D status and lung function was found [4,5,40], while in others this was not confirmed [6,7]. Two studies showed an association between serum 25(OH)D concentrations and lung function in patients with COPD and asthma [11,12]. These differences in study results might be caused by differences in study population and vitamin D status of participants. In our study, we observed an association in participants with obesity, in addition to a dose–response trend after stratification by BMI categories. This is in line with the finding of a previous study that observed an association between 25(OH)D and lung function in participants with obesity, but not in those without obesity [27]. Another recent study in asthmatic children reported an association between vitamin D status and lung function in obese, but not in non-obese children [41].

The explanation for this relationship in obesity only is unclear. It remains difficult to disentangle true causal associations in obesity from the potential confounding effect of obesity as a common cause of both low vitamin D concentrations and impaired lung function. One explanation might be that a potential relationship of vitamin D status with lung function is only present in participants with vitamin D deficiency. As vitamin D deficiency is more prevalent in individuals with obesity, this might explain why we did find a relationship in obese, but not in non-obese participants. A sensitivity analysis in participants with vitamin D deficiency (serum 25(OH)D concentrations < 50 nmol/L) in our study did not show an association. However, this group was small and might therefore have been underpowered.

Several studies have shown an association between adiposity and lung function [42,43,44,45]. A potential effect of obesity on lung function has been explained by effects on metabolic dysregulation, systemic inflammation and mechanical load of truncal fat [46]. Nevertheless, vitamin D deficiency has also been associated with recent-onset obesity [47,48] and it has been hypothesized that adiposity is an intermediate in the relationship between vitamin D and lung function through a direct mechanical effect on the diaphragm [27]. Finally, an explanation for the findings might be that adiposity is indeed an effect modifier. Vitamin D might exert a direct effect on lung function through tissue remodeling, muscle function and/or airway inflammation and the presence of adiposity may affect this relationship, as recently suggested [13].

We also observed an association between serum 25(OH)D concentrations and Fe_NO_ in obese, but not in non-obese participants. Fe_NO_ is a marker for Th2-mediated allergic inflammation, which is mainly used in the diagnostics of allergic asthma. Fe_NO_ is produced by lung epithelial cells mainly by inducible nitric oxide synthase (iNOS/*NOS2*), of which expression is increased during allergic airway inflammation [49,50]. In our study, we found a negative relationship between serum 25(OH)D concentrations and Fe_NO_ levels in obese individuals. This suggests that a higher vitamin D status is associated with lower airway inflammation in these participants. Vitamin D is known to be a potent immunomodulator and has been shown to decrease inflammatory reactions in vitro [15,51]. Few studies, however, have investigated the relationship between vitamin D and airway inflammation. In two cross-sectional studies in (asthmatic) children, serum 25(OH)D concentrations were not associated with Fe_NO_ [52,53]. Three intervention trials did also not show an effect of vitamin D supplementation on Fe_NO_ in patients with asthma [54,55,56]. Potential differences with our findings might be caused by the differences in study design. Previous studies did not report on potential effect modification of obesity. In one study, obese individuals were even excluded from the study [53]. In addition, our study was performed in the general adult population. Fe_NO_ levels are likely to be lower in the general population than in preselected cohorts of asthma patients. Possibly, vitamin D suppresses the lower levels of iNOS expression in these populations more readily than in patients with eosinophilic asthma. However, this is a hypothesis and exact mechanisms remain to be elucidated in future studies.

In our study, serum 25(OH)D concentrations were not related to the occurrence of a common cold in the preceding month. The relationship between vitamin D and respiratory infections has been extensively studied in observational studies [8,57]. In addition, trials assessing the effect of vitamin D supplementation have remained inconclusive [58,59,60,61,62]. The most recent meta-analysis showed a protective effect of vitamin D supplementation against respiratory infections, with larger effects in patients with vitamin D deficiency at baseline [62]. In our study, only 20% of the study population had serum 25(OH)D concentrations less than 50 nmol/L. This might explain why we did not find an association with the occurrence of a common cold in our study.

Strengths of this study are the large study population and the detailed phenotyping of the population, allowing extensive adjustment for relevant confounding factors. A major limitation of this study is the cross-sectional design. Therefore, no conclusions can be drawn regarding the direction and causality of relationships. Another limitation is that serum 25(OH)D measurements were performed by three different assays during the study period. To minimize possible variations, we calibrated our serum 25(OH)D measurements to the golden standard LC-MS/MS. In addition, the occurrence of a common cold, medical history and medication use were assessed by self-report, which might affect reliability. This may have resulted in an underestimation of the occurrence of common colds, which might have led to a reduced power to detect a potential relationship. We did not have reliable data on vitamin D supplementation use and dietary intake, and therefore could not rule out an effect of vitamin D supplementation. In addition, we did not have data on sunshine exposure. The physical activity questionnaire, however, did contain several outdoor activities such as cycling, walking and gardening, and, therefore, could be used as a proxy for sunshine exposure. Finally, while we did find associations of serum 25(OH)D concentrations with lung function and airway inflammation in obese participants, these differences were very small. This makes it difficult to assess the clinical relevance of our results.

## 5. Conclusions

This study showed that higher serum 25(OH)D concentrations were associated with a better lung function and less airway inflammation in obese, but not in non-obese individuals. Serum 25(OH)D concentrations were not associated with the occurrence of a recent common cold. Further studies are needed to assess the causal pathways and clinical relevance of our findings. Studies investigating the effects of vitamin D supplementation should specifically target persons with obesity and study specific effects stratified by BMI. Finally the underlying mechanisms by which obesity affects the relationship of serum 25(OH)D is still unclear and needs to be further studied.

## Figures and Tables

**Table 1:** Characteristics of participants aged 45–65 years of the Netherlands Epidemiology of Obesity study, stratified by serum 25(OH)D (nmol/L) category.

25(OH)D (nmol/L) Category	<50	50–75	≥75	*p*-Value *
Proportion of study population (%)	20	37	43	
25(OH)D (nmol/L)	39.5 (8.6)	62.7 (7.2)	93.6 (14.7)	<0.01
Age (years)	55.0 (6.7)	55.9 (6.1)	55.7 (5.6)	0.05
Sex (% men)	47	48	39	<0.01
White (%)	87	97	98	<0.01
Educational level (%high)	45	47	48	0.31
Smoking (%)				
Current	20	17	13	<0.01
Former	40	45	49	<0.01
Packyears	2.9 (0.0–15.2)	3.6 (0.0–15.2)	2.2 (0.0–14.0)	0.12
Season (% winter)	66	55	38	<0.01
Physical activity (MET/h)	23.0 (11.0–44.3)	28.5 (15.0–46.8)	34.5 (19.5–55.9)	<0.01
BMI (kg/m^2^)	27.4 (5.9)	26.5 (4.4)	25.5 (3.6)	<0.01
Total body fat (%)	32.3 (10.4)	31.5 (9.1)	31.3 (7.5)	0.03
Waist circumference (cm)	94.9 (16.6)	93.2 (13.1)	89.8 (11.7)	<0.01
Self-reported asthma (%)	6.1	4.8	3.7	0.02
Self-reported COPD (%)	5.4	4.7	3.3	<0.01
Use of pulmonary and anti-inflammatory medication (%)	15	13	13	0.11
FEV_1_ (%predicted)	105.7 (18.8)	107.2 (16.8)	109.3 (14.1)	<0.01
FVC (%predicted)	113.4 (17.7)	115.9 (16.9)	118.8 (14.3)	<0.01
Fe_NO_ (ppb)	19.0 (14.5)	19.0 (13.5)	18.7 (11.0)	0.57
Self-reported common cold in preceding month (%)	28	22	21	<0.01

Data are presented as mean (SD), percentage or median (interquartile range). Results are based on analyses weighted towards the BMI distribution of the general population (*n* = 6138). 25(OH)D: 25-hydroxyvitamin D; BMI: Body Mass Index; COPD: Chronic Obstructive Pulmonary Disease; FEV_1_: Forced Expiratory Volume in 1 s. FVC: Forced Vital Capacity; Fe_NO_: fractional exhaled nitric oxide; ppb: parts per billion. * *p*-value for trend.

**Table 2:** Associations of serum 25(OH)D (per 10 nmol/L) with FEV_1_, FVC, Fe_NO_ and occurrence of a common cold in men and women participating in the Netherlands Epidemiology of Obesity study, aged between 45 and 65 years.

	Crude	Multivariate ^1^	+BMI, TBF, WC ^2^
	Regression coefficient (95% CI) per 10 nmol/L 25(OH)D		
FEV_1_ (% predicted)	0.48 (0.23 to 0.73)	0.23 (−0.05 to 0.51)	0.10 (−0.18 to 0.39)
FVC (% predicted)	0.83 (0.58 to 1.07)	0.51 (0.24 to 0.77)	0.31 (0.04 to 0.57)
Fe_NO_ (ppb)	−0.18 (−0.39 to 0.03)	0.15 (−0.07 to 0.38)	0.16 (−0.06 to 0.36)
	Odds Ratio (95%CI) per 10 nmol/L 25(OH)D		
Common cold	0.94 (0.90 to 0.98)	1.00 (0.95 to 1.04)	1.00 (0.96 to 1.05)

Results were based on analyses weighted towards the BMI distribution of the general population (*n* = 6138), and were derived from regression coefficients with 95% confidence intervals from linear regression analyses and expressed as difference in outcome measure per 10 nmol/L 25(OH)D. ^1^ Multivariate: Adjusted for age, sex, ethnicity, number of packyears, self-reported obstructive pulmonary disease, use of pulmonary and anti-inflammatory medication, educational level, season and physical activity. ^2^ Multivariate plus adjustments for BMI, total body fat and waist circumference. CI: confidence interval; BMI: body mass index; FEV_1_: Forced Expiratory Volume in 1 s; FVC: Forced Vital Capacity; Fe_NO_: fractional exhaled nitric oxide; ppb: parts per billion; OR: Odds Ratio.

## Supplementary Materials

The following are available online at www.mdpi.com/2072-6643/10/1/35/s1, Table S1: Associations of serum 25(OH)D with FEV_1_, FVC, Fe_NO_ and occurrence of a common cold in participants with 25(OH)D concentrations <50 nmol/L; Table S2: Associations of serum 25(OH)D (per 10 nmol/L) with FEV1, FVC, FeNO and presence of a common cold in men and women participating in the Netherlands Epidemiology of Obesity study using vitamin D and multivitamin supplements; Table S3: Associations of serum 25(OH)D (per 10 nmol/L) with FEV_1_, FVC, Fe_NO_ and presence of a common cold stratified by BMI category, in men and women participating in the Netherlands Epidemiology of Obesity study, aged between 45 and 65 years; Table S4: Crude associations of serum 25(OH)D (per 10 nmol/L) with FEV_1_, FVC, Fe_NO_ and presence of a common cold stratified by age, in men and women participating in the Netherlands Epidemiology of Obesity study, aged between 45 and 65 years; Table S5: Crude associations of serum 25(OH)D (per 10 nmol/L) with FEV_1_, FVC, Fe_NO_ and presence of a common cold stratified by sex, in participants of the Netherlands Epidemiology of Obesity study, aged between 45 and 65 years.
