# Supplementary material for: Associations of Serum 25(OH)D Concentrations with Lung Function, Airway Inflammation and Common Cold in the General Population
Source: Nutrients. 2018 Jan 3;10(1):35. doi: 10.3390/nu10010035 (PMC5793263; doi:10.3390/nu10010035)
Supplement: Supplementary file 1 [file nutrients-10-00035-s001.pdf]

Table S1. Associations of serum 25(OH)D (per 10 nmol/L) with FEV_1, FVC, Fe_{NO} and presence of a common cold in men and women participating in the Netherlands Epidemiology of Obesity study with 25(OH)D levels < 50 nmol/L.

	**Crude**	**Multivariate[1]**	**+ BMI, TBF, WC[2]**
	**Regression coefficient (95% CI) per 10 nmol/L 25(OH)D**		
**FEV_1 (%predicted)**	0.98 (-1.02 to 2.98)	-0.39 (-2.33 to 1.56)	-0.55 (-2.50 to 1.39)
**FVC (%predicted)**	2.12 (0.35 to 3.88)	0.52 (-1.16 to 2.20)	0.35 (-1.21 to 1.91)
**Fe_{NO} (ppb)**	-0.60 (-1.88 to 0.67)	-0.35 (-1.45 to 0.76)	-0.08 (-1.19 to 1.03)
	**Odds Ratio's (95%CI) per 10 nmol/L 25(OH D**		
**Common cold**	0.79 (0.64 to 0.98)	0.83 (0.66 to 1.04)	0.83 (0.66 to 1.04)

Results were based on analyses in a subset of participants with 25(OH)D levels<50 nmol/L, weighted towards the BMI distribution of the general population (n=1498), and were derived from regression coefficients with 95% confidence intervals from linear regression analyses and expressed as difference in outcome measure per 10 nmol/L 25(OH)D. [1] Multivariate: Adjusted for age, sex, ethnicity, number of packyears, self-reported obstructive pulmonary disease, season, use of pulmonary and anti-inflammatory medication, educational level and physical activity. [2] Multivariate plus adjustments for BMI, total body fat and waist circumference.

BMI: Body mass index; OR: Odds Ratio. FEV_1: Forced Expiratory Volume in 1 s; FVC Forced Vital Capacity; Fe_{NO}: fractional exhaled nitric oxide; ppb: parts per billion; OR: Odds Ratio.

Table S2. Associations of serum 25(OH)D (per 10 nmol/L) with FEV_1, FVC, Fe_{NO} and presence of a common cold in men and women participating in the Netherlands Epidemiology of Obesity study using vitamin D and multivitamin supplements.

	**Crude**	**Multivariate[1]**	**+ BMI, TBF, WC[2]**
	**Regression coefficient (95% CI) per 10 nmol/L 25(OH)D**		
**FEV_1 (%predicted)**	0.34 (-0.13 to 0.80)	0.09 (-0.41 to 0.59)	0.05 (-0.40 to 0.55)
**FVC (%predicted)**	0.73 (0.26 to 1.21)	0.46 (-0.05 to 0.98)	0.35 (-0.16 to 0.87)
**Fe_{NO} (ppb)**	-0.49 (-1.05 to 1.07)	-0.15 (-0.65 to 0.35)	-0.14 (-0.64 to 0.35)
	**Odds Ratio's (95%CI) per 10 nmol/L 25(OH D**		
**Common cold**	0.94 (0.87 to 1.01)	0.97 (0.89 to 1.05)	0.98 (0.90 to 1.06)

Results were based on analyses in a subset of participants using vitamin D and multivitamin supplements, weighted towards the BMI distribution of the general population (n=1461), and were derived from regression coefficients with 95% confidence intervals from linear regression analyses and expressed as difference in outcome measure per 10 nmol/L 25(OH)D. [1] Multivariate: Adjusted for age, sex, ethnicity, number of packyears, self-reported obstructive pulmonary disease, season, use of pulmonary and anti-inflammatory medication, educational level and physical activity. [2] Multivariate plus adjustments for BMI, total body fat and waist circumference.

BMI: Body mass index; OR: Odds Ratio. FEV_1: Forced Expiratory Volume in 1 s; FVC Forced Vital Capacity; Fe_{NO}: fractional exhaled nitric oxide; ppb: parts per billion; OR: Odds Ratio.

Table S3. Associations of serum 25(OH)D (per 10 nmol/L) with FEV_1, FVC, Fe_{NO} and presence of a common cold stratified by BMI category, in men and women participating in the Netherlands Epidemiology of Obesity study, aged between 45 and 65 years.

		**Crude**	**Multivariate[1]**	**+ BMI, TBF, WC[2]**
		**Regression coefficient (95%CI) per 10 nmol/L 25(OH)D**		
FEV_1%	BMI <25	0.06 (-0.42 to 0.54)	-0.09 (-0.63 to 0.45)	-0.10 (-0.61 to 0.42)
	BMI 25-30	0.46 (0.14 to 0.78)	0.24 (-0.11 to 0.59)	0.12 (-0.23 to 0.46)
	BMI ≥30	0.70 (0.44 to 0.97)	0.69 (0.39 to 0.99)	0.46 (0.17 to 0.75)
FVC%	BMI <25	0.39 (-0.08 to 0.86)	0.09 (-0.42 to 0.59)	0.10 (-0.39 to 0.60)
	BMI 25-30	0.68 (0.37 to 0.98)	0.50 (0.18 to 0.82)	0.36 (0.05 to 0.67)
	BMI ≥30	0.88 (0.61 to 1.14)	0.72 (0.42 to 1.01)	0.46 (0.18 to 0.74)
Fe_{NO} (ppb)	BMI <25	-0.24 (-0.65 to 0.18)	0.21 (-0.22 to 0.63)	0.23 (-0.19 to 0.65)
	BMI 25-30	-0.15 (-0.43 to 0.12)	0.14 (-0.16 to 0.44)	0.14 (-0.16 to 0.44)
	BMI ≥30	-0.36 (-0.53 to -0.19)	-0.24 (-0.42 to -0.05)	-0.24 (-0.43 to -0.04)
		**Odds Ratio's per 10 nmol/L 25(OH)D**		
Common cold	BMI <25	0.95 (0.88 to 1.02)	1.02 (0.94 to 1.10)	1.02 (0.94 to 1.11)
	BMI 25-30	0.96 (0.91 to 1.01)	1.00 (0.94 to 1.06)	1.00 (0.94 to 1.06)
	BMI ≥30	0.89 (0.85 to 0.93)	0.96 (0.91 to 1.01)	0.97 (0.92 to 1.01)

Results were based on analyses weighted towards the BMI distribution of the general population (n=6138), and were derived from regression coefficients with 95% confidence intervals from linear regression analyses and expressed as difference in outcome measure per 10 nmol/L 25(OH)D stratified by BMI-category (BMI<25: 43%, BMI 25 -30: 41% and BMI ≥30: 16%). [1] Multivariate: Adjusted for age, sex, ethnicity, number of packyears, self-reported obstructive pulmonary disease, use of pulmonary and anti-inflammatory medication, educational level, season and physical activity. [2] Multivariate plus adjustments for BMI, total body fat and waist circumference. FEV_1: Forced Expiratory Volume in 1 s; FVC: Forced Vital Capacity; Fe_{NO}: fractional exhaled nitric oxide; ppb: parts per billion; BMI: Body Mass Index.

Table S4. Crude associations of serum 25(OH)D (per 10 nmol/L) with FEV_1, FVC, Fe_{NO} and presence of a common cold stratified by age, in men and women participating in the Netherlands Epidemiology of Obesity study, aged between 45 and 65 years.

	**<50 years**	**50-55 years**	**55-60 years**	**>60 years**
	**Regression coefficient (95% CI) per 10 nmol/L 25(OH)D**			
**FEV_1 (%predicted)**	0.55 (0.08 to1.02)	0.69 (0.15 to 1.23)	0.32 (-0.24 to 0.87)	0.38 (-0.06 to 0.82)
**FVC (%predicted)**	0.84 (0.36 to 1.32)	0.99 (0.46 to 1.52)	1.03 (0.52 to 1.54)	0.57 (0.11 to 1.02)
**Fe_{NO} (ppb)**	-0.30 (-0.84 to 0.24)	-0.43 (-0.87 to 0.00)	-0.05 (-0.37 to 0.28)	-0.04 (-0.40 to 0.32)
	**Odds Ratio's (95%CI) per 10 nmol/L 25(OH)D**			
**Common cold**	0.91 (0.84 to 0.99)	0.90 (0.83 to 0.98)	1.00 (0.93 to 1.07)	0.94 (0.88 to 1.01)

Results were based on analyses weighted towards the BMI distribution of the general population (n=6138), and were derived from regression coefficients with 95% confidence intervals from linear regression analyses and expressed as difference in outcome measure per 10 nmol/L 25(OH)D stratified by age-category (age <50: 21%, 50-55:22%, 55-60: 23% and ≥60: 34%).FEV_1: Forced Expiratory Volume in 1 s; FVC: Forced Vital Capacity; Fe_{NO}: fractional exhaled nitric oxide; ppb: parts per billion; BMI: Body Mass Index.

Table S5. Crude associations of serum 25(OH)D (per 10 nmol/L) with FEV_1, FVC, Fe_{NO} and presence of a common cold stratified by sex, in participants of the Netherlands Epidemiology of Obesity study, aged between 45 and 65 years.

	**Men**	**Women**
	**Regression coefficient (95% CI) per 10 nmol/L 25(OH)D**	
**FEV_1 (%predicted)**	0.54 (0.16 to 0.92)	0.33 (0.01 to 0.65)
**FVC (%predicted)**	0.56 (0.23 to 0.89)	0.72 (0.39 to 1.04)
**Fe_{NO} (ppb)**	0.00 (-0.36 to 0.36)	-0.17 (-0.42 to 0.07)
	**Regression coefficient (95% CI) per 10 nmol/L 25(OH)D**	
**Common cold**	0.92 (0.87 to 0.97)	0.96 (0.91 to 1.01)

Results were based on analyses weighted towards the BMI distribution of the general population (n=6138), and were derived from regression coefficients with 95% confidence intervals from linear regression analyses and expressed as difference in outcome measure per 10 nmol/L 25(OH)D stratified by sex (men: 44%, women: 56%). FEV_1: Forced Expiratory Volume in 1 s; FVC: Forced Vital Capacity; Fe_{NO}: fractional exhaled nitric oxide; ppb: parts per billion; BMI: Body Mass Index.
